# Etiology of Inebriety

**Published:** 1881-11

**Authors:** T. D. Crothers

**Affiliations:** Supt. Walnut Lodge, Hartford, Conn.


### ARTICLE IV.

ETIOLOGY OF INEBRIETY. By T. D. CROTHERS, M.D., Supt. Walnut Lodge, Hartford, Conn.

The acceptance of every new truth in medicine affecting the interest of the masses, is through the ordeal of ignorant opposition and criticism. The recognition and acceptance of the truth that insanity is a disease, was over half a century passing this period. Yet within a year the same ignorant criticism has appeared in several newspapers, urging that the insane should be held personally responsible, and punished for their acts, and not housed up and treated as sick men. The truths of the disease of inebriety are passing through the same stage. The most confusing theories, and absurd assumptions and denials, not based on any reliable studies or facts, prevail. The study of inebriety is yet in its infancy, and it is impossible from any experience or study at this time, to do more than outline some general landmarks, which stand out prominently above the wide ranges of hill and valley entirely unknown. I propose to group some of the general facts which have been noted by many observers, in the field of causes, for the purpose of showing, not how much is known, but rather the vast continent as yet unexplored, on the borders of which scientific inquiry is gathering to enter this realm, and point out the truths of this almost *Central Africa* of medical and social science.

Every case of inebriety is marked by some cerebral defect, and enfeebled will-power, with perverted nutritive functions which begins back in some special condition or event, effecting both the structure and function of the organism. Every case will be found to follow a certain progressive march from stage to stage, with long obscure halts, or rapid strides to chronicity and death. Both exciting and predisposing causes are at work; the one may produce changes of functions and structure, which may bring on disease, or awaken latent tendencies to it; the other may induce such a state of the system inducing this disease, or weakening the power of resisting all morbific agencies which end in this malady. As in all diseases the causes all run into each other, often beginning in the organism, and in other cases in the mind, ranging over the entire field of mental pathology and closely allied with all forms of insanity. The causes divide most naturally into three classes or groups: *First.* Causes which come from inheritance, direct or indirect. *Second.* Such general causes followed by inebriety in common with insanity. *Third.* Special conditions and circumstances which favor the development of inebriety.

*First.*—Hereditary influence. No other disease is more certainly transmitted, not in the desire, always, for alcohol or its compounds, but in nerve degenerations and perversions, which sooner or later break out in inebriety depending on some obscure conditions. The very poor and the very wealthy furnish the most striking illustrations of this fact. The former who not only suffer from irregularities of hunger and satiety, with bad quality and conditions of food, but perverted nutrition and diminished vitality, always transmit inebriate tendencies; the latter through continuous stimulation and excess, with neglect of healthy activity of both body and mind, develop nutritive degenerations both functional and organic, which appear in the next generation in inebriety. Both occupation and mental condition at the time of procreation are active factors in the causation. Persons who are engaged in intense mental work, and leading a sedentary life, such as literary men, clergymen, bankers, book-keepers, etc., have often inebriate descendants. Inebriety not only follows from inebriate parents, but from excitable nervous persons, who have an unstable mental organization, with disordered emotional faculties, and unbalanced nutritive functions manifest often in a capricious irregular appetite for unusual foods and fluids, etc. An inebriate diathesis can be noted in many cases, which from the absence of some special exciting causes do not always develop into inebriety. This can be studied in the practice of any physician, for there are many persons in every community who possess all the latent tendencies to inebriety; nothing but exposure to some peculiar exciting causes prevents them from being inebriates. Not unfrequently persons discover this peculiar diathesis, and avoid with great care all temptation, or conditions of surroundings which encourage the use of spirits. This diathesis is not unfrequently associated with general organic degeneration, marked by anæmia, neuralgia, affections of the heart, rheumatism, dyspepsia, tubercular deposits, and constant tendency to exhaustion, etc. The intellect is active, but uncertain, changeable, vain, sometimes with the force of a genius, and the weakness of a child, but always emotional, sensitive and sympathetic. The body and mind are developed out of proportion, unnatural special developments are present, the functional activity is intense, and they are either on the verge of eccentricity, or abject feebleness.

*Second.*—Such general causes from which both inebriety or insanity may follow. Of these traumatism or positive injury to the brain, or spinal cord, has been noted in many cases. These injuries are followed by alterations of nutrition, exhaustion, physical pain and depression, for which alcohol acts as a pure sedative and is taken as food, is used to relieve the cravings of hunger. Blows on the head, and injuries to the nervous trunks are often followed by inebriety. A previously temperate man falls striking on his head, or receives a blow which is soon forgotten, but he becomes a different man, and an inebriate without any reason or cause that can be seen by his friends. I have noted many cases of inebriety from railroad accidents, where the concussion and alarm were sudden, causing intense reaction and excitement, and yet no physical lesion was apparent. Injuries to distant parts of the body, react by some unknown law in inebriety. A chaplain in the late war was injured in the leg, and drank spirits to intoxication for two years until the wound healed, then became a temperate man. A physician was thrown from his carriage, suffering from a broken arm, and became an inebriate at once. Many months after he reformed, dating from the healing of the injury. Four instances have been noted where the expulsion of a tape-worm was followed by complete recovery from the inebriety, indicating the influence of this as a cause. Other cases have been noted in which irritation from uterine troubles, and disturbances of the genital functions have appeared in inebriety. During pregnancy and lactation excessive craving for alcohol is not uncommon. There is a field of causes along the line of traumatism which, in my opinion, will throw great light on the obscure cases, and explain much that is now unknown. Conditions of degeneration and impairment of functions, either inherited or acquired may exist, which break out in inebriety either gradually or suddenly, from a train of causes that are inadequate to explain such results. The brain and nervous system has lost some power of restoration by which its integrity is preserved, or it has taken on certain conditions favorable to the development of inebriety or insanity. This may be illustrated in the physical condition following a severe attack of pneumonia; the lung power and function of respiration is defective ever after, although it cannot be defined. After an attack of typhoid fever, the patient is fully aware of impairment of force, and a want of something which existed before, although his recovery seems complete. Injury to the brain, or severe organic maladies, leave an entailment which influences the organism and mind after. Sunstroke, the toxic action of alcohol or opium, paroxysms of grief, fear, alarm, joy, may have the same effect. The inherited defects may only appear in this form; epilepsy, insanity in all its many phases, and inebriety, all indicate a brain soil differing from that of a sound person. In all these cases there is something wanting to make up cerebral health, which can only be determined by careful study in the future. I have long been convinced that the results of previous diseases were active causes in inebriety. Intermittent and malarious fevers, acute rheumatism, many of the diseases of the stomach, liver and kidneys, will be found to end in inebriety. Persistent neuralgias often break up the mind-centers, and produce exhaustion for which alcohol is found to be almost a panacea, and inebriety follows with great certainty.

Dietetic disturbances are frequently associated with the beginning of inebriety. This is apparent in the close relation of the nutritive functions and the nervous system. Dyspepsia, with all its train of perverted sensations and tastes, passes most naturally into inebriety. Excessive hunger and thirst is followed by conditions which end in inebriety. Practically any disease which breaks up the nutritive functions, causes perversions and cerebral disturbances, predisposes to inebriety in many cases. The history of inebriety is very often one of neglect of proper nutrition and general hygienic care at the beginning, then the use of beer and stronger alcohols follow. Exhaustive intellectual or physical exertion, develops either inebriety or some other form of cerebral disturbance. Scholars and students often exhibit strong tastes and longings, indicating an unbalanced mind and body, on the verge of inebriety. A prominent senator spent thirty hours in the preparation of a speech, and was an inebriate from this time; the proprietor of an ice-house tempted his workmen to continue work over forty hours continuously without much rest; two out of five men became inebriates from this occasion. A clergyman who worked excessively in a revival for many weeks was an inebriate from this time. A farmer who was overworking in the harvest field for many days drank large quantities of ice water, suffered from colic, then drank and died of inebriety two years after. Loss of property, disappointment, social or financial, grief, religious excitement, an unbalanced mind and misdirected education, are all active causes entering into the origin of inebriety. Many things in our systems of education lead directly to this end. The unphysiological methods of teaching, in bad surroundings, are the fertile soils for perverted tastes, and ignorant eccentricities from which both mental and physical dyspepsia follow; then the door is open for inebriety or some of the insanities. The dangerous ambition to sacrifice everything for wealth, power or place breaks up all healthy living, and inebriety is the result of both failure and success. Any special train of causes which break up the normal activity of the body is followed by exhaustion, and a craving for means to build up against this loss. Often this is manifest in nutrient desires, or nervous depres sion. For both these states alcohol in any form, either as bitters or in beer, acts as a sedative, and inebriety is thus begun.

*Third.*—*Special* conditions and circumstances which favor the development of inebriety, and which are noted in the majority of cases. Of these, certain periods or ages are more dangerous than others. This is founded on the physiological eras in the organization, or stages of development and decline, which have pathological relations that influence both the nervous and nutrient system. The first most dangerous period is puberty. Prolonged sexual excitement at this time is the beginning of inebriety which may not break out until full manhood. Intoxication after sexual intercourse at this period, powerfully impresses the organism with a distinct predisposition to inebriety. Later in adult life when the struggle is at its height, and all the faculties are intensely occupied, the ambitions, disappointments and changes are sudden and far-reaching; when confidence in the physical and mental powers of recovery cause recklessness of effects, then the body is extremely susceptible to inebriety and conditions which develop this malady. At this midland station between the weakness of infancy and the decline of old age, when the brain and nervous system has reached its fullest activity, both the inherited and exciting causes may concentrate in this disorder. Dr. L. D. Mason, of Fort Hamilton, N. Y., found these periods of greatest danger to be from fifteen to twenty-five, and from thirty to thirty-five.

Surroundings have a very potent influence in the production of inebriety. Mental states that give no time for change or rest, with monotonous tread-mill thoughts over and over again, as light-house keepers, men on the frontier, farmers far back in the interior, workmen who are closely confined to some special circle, telegraph operators, etc., where for years the same unchanging mental conditions exist. Or, on the other, between continuous change and strain to accommodate the mind to the ever-shifting scenes that appeal to the reason and emotional faculties: as, for instance, speculators, railroad and professional men, who are occupied a large part of the time, without chance for rest and change of thought. The influence of social surroundings which are degenerating and dwarfing of all the better elements of nature, are dangerous. Low society and company follows either after inebriety has begun, or is associated with a defective brain and enfeebled moral power before alcohol is used. Domestic troubles, changing conditions of life, stimulated by pride or ambition, all help to break up the normal state of health. How far these and other circumstances of a social character enter into the causation of inebriety is not determined; but it is clear that the mind and the condition of the nervous system are very large factors in inebriety not now recognized.

The physical surroundings have a very potent influence in these cases. Temperature is often an active cause. High degrees of heat and cold may so far impair the nutrition of the nerves as to produce paresis of the vaso-motor centers, ending in inebriety. Men in profuse perspiration suddenly cooling off have become inebriates. Firemen exposed to great heat often suffer in this way. Men employed on the *South American steamers*, that ply between New York, Boston and the tropical ports, where the extremes of climate, heat and cold, alternate in rapid succession, from the frequent polar temperature of New York, to the summer tropics, soon become inebriates. Regularity and equality of food have great influence in the causation. The moment the proper nutrition of the body, both as to time and quality of food is disturbed, the soil is made ready for inebriety. Eating at irregular times, as among business and professional men who are closely engaged in absorbing work, brings on nutritive perversions; and when the character of the food is imperfect for the nourishment of the body, still further departures from health follow. If with these factors are associated unhealthy sleeping rooms, and places of business, bad air and damp unpleasant surroundings, a train of causes are at work of which inebriety is almost certain to follow. The disturbed nutrition of the body, followed by conditions of neurasthenia precedes inebriety, and every unsanitary condition intensifies and fixes this state. Certain kinds of labor, in bad surroundings and living, are also factors tending towards this malady. As, for instance, tobacco-raising in certain river bottoms in the South-west; coal mining in particular districts of England and Pennsylvania; and some kinds of manufacturing in the eastern states, etc. There are, in these, and other physical states, combinations of causes unknown at present, where inebriety, pauperism insanity, and crime often seem to flourish indigenous. There are many forces which govern the wants of the organism, that cannot be understood to-day. The influences of the mountain, or sea air, of the effect of the winds, and degrees of heat and cold, the presence of storms and electrical changes in the air, are all noted in those who suffer from mental diseases, and among inebriates ; but how far they enter into the early causation, the student of the next century must determine. In the reports of the early insane asylums, bad temper was put down as an active cause of insanity. Now this is understood as an early symptom of disease and departure from mental health. To ascribe the first cause of inebriety to sociability, association, bad company, etc., is equally an error. These are only accidental circumstances and coincidents, which, in a certain proportion of cases, both fix and explode a long train of causes, gathering from the past and present. The causes of inebriety must be sought farther back than the statement of the patient, or his friends. His entire life must be studied, and the evidence of heredity carefully weighed, and all the circumstances of his mental, physical, social, and sexual life examined. From this only can be ascertained the causes which are formative in one of the most widespread diseases of this age. Inebriety is undoubtedly associated either as a symptom or cause of insanity in many instances, but in the vast proportion of cases it stands out alone, as a distinct disease. The complex causes which govern its march, and the obscure influences which seem to select the men of genius and talent, and those representing the other extreme of mental capacity, hurrying them on to destruction ; the pathology and the symptomatology, and the prognosis and treatment, are all in an unknown realm. Standing on this border-land, the evidences of the domain of law rich in physiological fact are apparent. When an accurate study shall clear up the fog and superstition, and inebriety and its nature will not be a question of opinion or theory, then its physical and psychical origin will be understood. Stretching out from these outline hints of causes, the medical study of inebriety gives promise of a new era in science and civilization. Some of the facts made prominent may be stated thus :

1. Inebriety is a cerebro-psychical disease, beginning in some cerebral defect in alteration of function or structure.

2. The causes are numerous and complex, blending with each other, and depending upon many and unknown physical conditions, either inherited or acquired.

3. Its etiology must be studied from a physiological point of view, by medical men, and only from the observations of years, and the experience of many accurate observers, cañ it be understood.

